# Phase II study of anlotinib in combination with oxaliplatin and capecitabine for patients with RAS/BRAF wild-type metastatic colorectal adenocarcinoma as the first-line therapy

**DOI:** 10.1186/s12916-022-02357-6

**Published:** 2022-05-06

**Authors:** Yue Liu, Qian Xiao, Jinjie He, Hanguang Hu, Jinlin Du, Yuping Zhu, Jiaqi Chen, Zhuo Liu, Jianping Wang, Lifeng Sun, Dong Xu, Jun Li, Xiujun Liao, Jianwei Wang, Yibo Cai, Cheng Cai, Zhekang Jin, Liuhong Wang, Ying Yuan, Kefeng Ding

**Affiliations:** 1 Colorectal Surgery and Oncology, Key Laboratory of Cancer Prevention and Intervention, Ministry of Education, The Second Affiliated Hospital, Zhejiang University School of Medicine, Hangzhou, China; 2 Medical Oncology, The Second Affiliated Hospital of Zhejiang University School of Medicine, Hangzhou, China; 3 Colorectal Surgery, Zhejiang University Jinhua Hospital, Jinhua, China; 4 Colorectal Surgery, Cancer Hospital of the University of Chinese Academy of Sciences (Zhejiang Cancer Hospital), Hangzhou, China; 5 Radiology, The Second Affiliated Hospital of Zhejiang University School of Medicine, Hangzhou, China; 6 Cancer Center Zhejiang University, Zhejiang, China

**Keywords:** Metastatic colorectal cancer, Anlotinib, Capecitabine, Oxaliplatin, First-line therapy

## Abstract

**Background:**

Anlotinib, an oral small molecule tyrosine kinase inhibitor targeting VEGFR 1/2/3, FGFR 1-4, PDGFR a/β, and c-kit, had demonstrated prolonged progression-free survival (PFS) in refractory metastatic colorectal cancer (mCRC). This multicenter, single-arm, phase II, exploratory study was conducted to evaluate the efficacy and safety of anlotinib combined with capecitabine and oxaliplatin as first-line treatment for unresectable RAS/BRAF wild-type mCRC.

**Methods:**

Patients aged 18–75 with RAS/BRAF wild-type unresectable mCRC, without prior systemic treatment, and ECOG performance status ≤1 were enrolled. Eligible patients received capecitabine (850 mg/m^2^, p.o., bid, on day 1–14 every 21 days), oxaliplatin (130 mg/m^2^, i.v., on day 1 every 21 days), and anlotinib (12 mg, p.o., qd, on days 1–14 every 21 days) as induction therapy. Following 6 cycles of therapy, patients who achieved response or stable disease received capecitabine and anlotinib as maintenance therapy until tumor progression. The primary endpoint was objective response rate (ORR) according to RECIST (version: 1.1), and the secondary endpoints were PFS, disease control rate (DCR), duration of response (DOR), and safety.

**Results:**

Between November 2019 and February 2021, 31 patients were enrolled. One patient was excluded for refusing treatment. The primary endpoint of ORR was 76.7% (95% CI, 57.7–90.1) with 1 patient achieving a complete response and 22 patients partial response. DCR was 93.3% (95% CI, 77.9–99.2). At a median follow-up of 14.1 months (95% CI, 9.9–18.3), median PFS was 11.3 months (95% CI, 7.1–14.1), and DOR was 7.9 months (95% CI, 5.5–12.7). Twenty-five (83.3%) patients experienced grade 3 or 4 treatment-emergent adverse events (TEAEs). No grade 5 TEAE was reported. The most common grade 3 or 4 TEAEs (>10%) were hypertension (15/30; 50%), neutrophil count decreased (8/30; 26.7%), and diarrhea (4/30; 13.3%). A total of 18 (60%) patients had TEAEs that resulted in dose reduction, interruptions, or delays.

**Conclusions:**

Anlotinib combined with capecitabine and oxaliplatin showed considerable ORR, DCR, PFS, and DOR in the first-line therapy of mCRC with manageable toxicity profiles.

**Trial registration:**

ClinicalTrials.gov: NCT04080843

## Background

Colorectal cancer (CRC) is the third most prevalent malignancy worldwide and is ranked as the second largest contributor to fatalities of patients after lung cancer. In 2020, more than 1.9 million CRC cases were diagnosed globally, with expected 935,000 deaths [1]. At the time of diagnosis, up to 20% of patients had metastatic disease. As the disease progressed, 40% of individuals with CRC had developed metastases [2]. In the case of metastatic colorectal cancer (mCRC), the prognosis is unsatisfied, with a 5-year survival rate of below 20% [3].

The conventional therapy for mCRC includes irinotecan, fluoropyrimidine, or oxaliplatin plus anti-vascular endothelial growth factor (VEGF) monoclonal antibodies or anti-epidermal growth factor receptor (EGFR) monoclonal antibodies, including bevacizumab, panitumumab, or cetuximab—based on Ras/Raf status. These treatment regimens significantly prolonged patients’ overall survival (OS) from 6 to ~20 months [4–7]. Immune therapy is used only for a small proportion of mCRC patients (4%) with microsatellite instability-high (MSI-H) or mismatch repair-deficient (dMMR) [8].

Anlotinib is an orally administered small molecule tyrosine kinase inhibitor (TKI) that targeted tyrosine kinases, including VEGF receptor 1/2/3, the fibroblast growth factor receptor (FGFR) 1–4, platelet-derived growth factor receptor (PDGFR) α/β, and c-Kit, and has a broad spectrum of inhibitory effects on tumor angiogenesis and growth [9, 10]. Clinical studies have confirmed the effectiveness of anlotinib in several advanced malignant cancers, including non-small cell lung cancer, small cell lung cancer, soft tissue sarcoma, medullary thyroid cancer, and renal cell carcinoma [11–15]. The phase III trial ALTER-0703 demonstrated the efficacy and tolerability of anlotinib monotherapy in mCRC patients who failed to achieve remission after standard treatment [16]. Anlotinib significantly prolonged the progression-free survival (PFS) of mCRC patients over placebo (4.1 months versus 1.5 months; HR = 0.34; *P* < 0.0001), while the median OS in the anlotinib group and placebo group was similar (8.6 months versus 7.2 months; HR = 1.02; *p* = .870). The subgroup analysis demonstrated the OS benefit of anlotinib in patients with KRAS/NRAS/BRAF wild-type mCRC (HR = 0.68, 0.47–0.99). Therefore, patients with RAS/BRAF wild-type mCRC might be potential candidates for anlotinib therapy.

A phase I/II study was conducted to investigate the safety and efficacy of anlotinib plus irinotecan in patients with advanced CRC who had received initial treatment. This trial demonstrated that the combination of anlotinib and irinotecan was a promising second-line therapy for advanced CRC with a manageable safety profile [17].

The safety and efficacy of anlotinib in combination with chemotherapy as a first-line treatment for mCRC have not been investigated previously as far as we know. The objective of the present study was to determine the efficacy and tolerability of anlotinib combined with oxaliplatin and capecitabine as a first-line therapy for RAS/BRAF wild-type mCRC.

## Methods

### Study design and participants

The ALTER-C-002 trial was a multicenter, single-arm, phase II, exploratory study (NCT04080843) conducted at 3 centers in Zhejiang, China. Each center had independent ethics committee that granted approval for the research protocol. The trial was performed in accordance with the principles of *Good Clinical Practice* and *Declaration of Helsinki* [18], as well as all relevant regulations and laws in the applicable countries. Before participating in the trial, all patients gave written informed consent.

Eligible patients should be aged between 18 and 75 with histologically or cytologically confirmed colon and rectum adenocarcinoma and RAS/BRAF wild-type. Patients were confirmed to have unresectable lesions or to have a metastatic disease without potentially resectable disease. RAS/BRAF wild-type mCRC patients who did not receive previous systemic therapy for mCRC or who received neoadjuvant/adjuvant chemotherapy for stages I–III CRC relapsed more than 6 months from the last administration of peri-operation chemotherapy were included. Other inclusion criteria were patients that had at least one measurable lesion according to Response Evaluation Criteria in Solid Tumors (RECIST) version 1.1 [19], Eastern Cooperative Oncology Group performance status of 0 or 1, adequate bone marrow, liver, and renal function, and a minimum of three-month predicted survival time duration. Primary exclusion criteria included previous treatment with anti-VEGF therapy or TKIs, and uncontrolled hypertension (systolic blood pressure >150 mm/Hg or diastolic blood pressure >90 mm/Hg) despite adequate care.

### Procedures

**Fig. 1:**
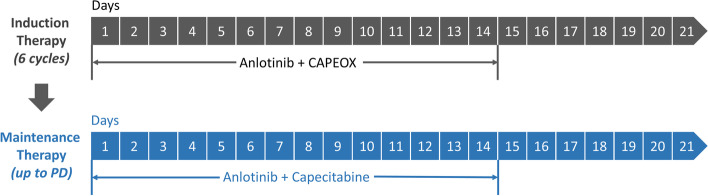
Therapy procedures (21-day cycle). Oxaliplatin:130mg/m^2^, ivgtt, d1; anlotinib: 12mg, po, qd, d1–14; capecitabine: 850mg/m^2^, po, bid, d1–14

Enrolled patients received capecitabine (850 mg/m^2^, p.o., bid, on days 1–14, every 21 days), oxaliplatin (130 mg/m^2^, i.v., on day 1, every 21 days), and anlotinib (12 mg, p.o., qd. on days 1–14, every 21 days). Patients who achieved response or stable disease (SD) were then administered with capecitabine and anlotinib as maintenance treatment after completing 6 cycles of induction therapy. An overview of the therapy procedures is shown in Fig. 1.

The medication was continued until tumor progression (according to RECIST version 1.1), unacceptable toxicity, withdraw of patients’ consent, death, or investigator’s decision to discontinue the treatment. Patients could continue to receive the remaining medications even if one of the drugs had to be discontinued due to toxicity. Dose modifications were conducted for any drug of this combination regimen in order to manage drug-related toxicities. Dosage of anlotinib could be reduced to 10 mg or even 8 mg. Dosage of oxaliplatin could be modified to 75% of the initial one, while that of capecitabine was allowed to be altered to 75% or even 50% of the initial one.

### Endpoints

The primary endpoint was objective response rate (ORR) based on investigator’s assessment according to RECIST version 1.1. ORR was defined as the proportion of patients achieved a best overall tumor response of complete response (CR) or partial response (PR). Secondary endpoints included PFS, disease control rate (DCR; refers to the proportion of patients with response and stable disease), duration of response (DOR), safety, and tolerability. PFS was defined as the time from the day when patients received the first dose of treatment regimen to the date of first documented progression or death from any cause, which ever firstly occurred, including induction and maintenance therapy. DOR was defined as the duration from the day when patients firstly had response to the day they had progressive disease. Severity of adverse events (AEs) was assessed according to the Common Terminology Criteria Adverse Events (NCI CTC AE version 4.03).

### Assessments

Tumor evaluation was performed by computed tomography or magnetic resonance imaging during the screening period and every two cycles throughout the study until disease progression. Survival evaluation was performed every 2 months until death or withdrawal of consent. Safety was recorded continuously until 30 days after the end of treatment.

### Statistical analysis

The objective of the study was to explore whether anlotinib combined with oxaliplatin and capecitabine could substantially enhance the ORR when compared to that of other studies of chemotherapies. The expected sample size was calculated according to the alternative hypothesis that the ORR with anlotinib plus oxaliplatin and capecitabine would be 61% or higher (H1=61%) and the null hypothesis that the ORR would be 31% or lower (H0=31%) [20–25]. With α of 5% and power of 90%, 27 cases would be recruited using the Clopper-Pearson method (PASS version 15, NSCC, LLC). Considering an approximate drop-out incidence of 10%, a total of 30 patients would be recruited.

The full analysis set (FAS) and safety analysis set (SAS), both of which comprised patients receiving a minimum of one dosage of anlotinib, were used to conduct the efficacy and safety evaluations, respectively. ORR and DCR were analyzed based on the Clopper-Pearson method. To assess the median value and 95% confidence interval (CI) of PFS, DOR, duration of treatment (DOT), duration of maintenance therapy, and follow-up duration, a Kaplan-Meier (KM) analysis was performed. All statistical analyses were carried out using SAS version 9.4 (SAS Institute Inc).

## Results

### Patient demographics and characteristics

Patient recruitment was initiated in November 2019 and ended in February 2021 when the desired number of patients was reached.

**Fig. 2:**
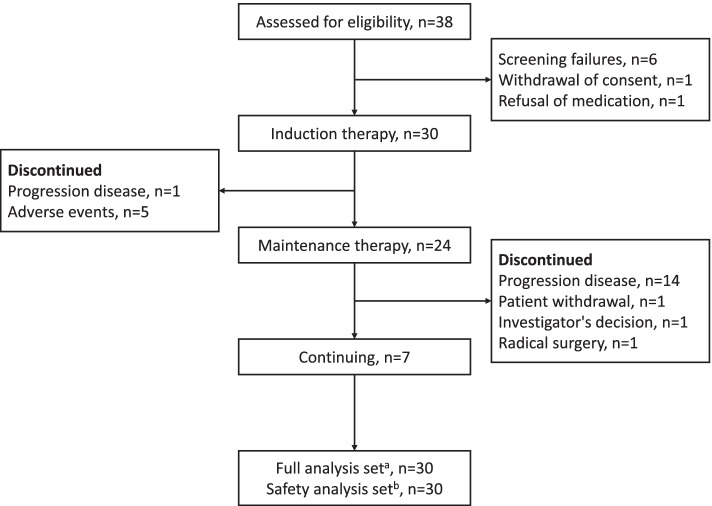
CONSORT diagram. 30 patients had ≥1 dose of the treatment regimen. ^b^ 30 patients received ≥1 dose of treatment regimen and safety had been recorded after administration. ^c^ The data cutoff date was October 15, 2021

Thirty eight patients were screened. Among these patients, 31 met the inclusion criteria with 1 patient excluded for refusing treatment. A total of 30 patients were included in FAS and SAS (Fig. 2). Patient demographic and baseline characteristics are presented in Table 1. Patients’ age ranged between 32 and 72 years with the median age being 60 years. The ratio of males to females was 26 (86.7%): 4 (13.3%). Three patients (10.0%) had an ECOG PS score of 0, while 27 patients (90.0%) had a score of 1. The left colon and rectum were found as primary tumor locations in 26 individuals (86.7% ). The majority of participants were found to have liver metastases (25; 83.3% ) and 25 patients (83.3%) had synchronous metastasis. Nineteen patients (63.3%) had primary tumors resected; among them, 5 received radical surgery.

**Table 1:** Patient demographic and baseline characteristics (full analysis set, *N*=30)

Characteristics	*n*	%
Male/female ratio	26/4	86.7:13.3
Age (years)		
Median (range)	60 (32–72)	
> 65	5	16.7
ECOG performance status		
0	3	10.0
1	27	90.0
TNM stage		
IV	30	100
CEA(ng/ml)		
< 5	2	6.7
5–200	16	53.3
> 200	10	33.3
Unknown	2	6.7
Primary disease site		
Colon	13	43.3
Rectum	16	53.3
Colon and rectum	1	3.3
Primary tumor location		
Right	4	13.3
Left colon and rectum	26	86.7
Primary tumor resected		
Yes	19	63.3
No	11	36.7
Metastatic sites		
Liver only	16	53.3
Liver + other	9	30
Other only	5	16.7
Metastatic status		
Synchronous	25	83.3
Metachronous	5	16.7
Diameter of the largest target lesions (mm)		
median (range)^a^	45 (16–122)	
MSI/MMR status^b^		
MSS/pMMR	25	83.3
MSI-H/dMMR	0	0
Unknow	5	16.7

*ECOG*, Eastern Cooperative Oncology Group; *TNM*, tumor, node, metastasis; *CEA*, carcinoembryonic antigen; *MSI*, micro satellite instability; *MMR*, mismatch repair

^a^The median is determined by IQR

^b^Thirteen patients received the MSI test by PCR and MMR test by immunohistochemistry, and 9 patients only received the MMR test by immunohistochemistry and 3 patients only received MSI test by PCR

Twenty-four patients who achieved response or SD after induction therapy subsequently received maintenance therapy. Seven patients were still treated with maintenance therapy at the data cut-off day (15 October 2021), and the median follow-up duration was 14.1 months (95% CI, 9.9–18.3).

### Efficacy

**Fig. 3:**
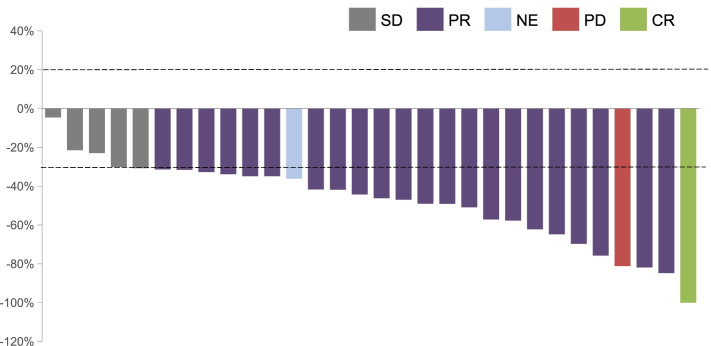
The best change in target lesion diameter from baseline (full analysis set, *N* = 30)

Of 30 patients in the FAS, a confirmed ORR was observed in 76.7% (95% CI, 57.7–90.1) of patients (23/30; 1 patient achieved confirmed CR and 22 patients achieved confirmed PR), while DCR was 93.3% (95% CI, 77.9–99.2); 1 patient had progressive disease (PD) in the second evaluation and 1 patient had inevaluable (NE) (PR in the first evaluation; however, the patient failed to take the second evaluation) (Table 2). All 30 patients had tumor shrank and the best change in target lesion diameter from baseline is shown in Fig. 3.

**Table 2:** Investigator-assessed response utilizing RECIST (version: 1.1)

Best overall response	Anlotinib + oxaliplatin + capecitabine (*n* = 30)
CR, *n* (%)	1 (3.3)
PR, *n* (%)	22 (73.3)
SD, *n* (%)	5 (16.7)
PD, *n* (%)	1 (3.3)
NE, *n* (%)	1 (3.3)
ORR^a^, *n* (%, 95%CI)	23 (76.7,57.7–90.1)
DCR^b^, *n* (%, 95%CI)	28 (93.3, 77.9–99.2)

*RECIST*, Response Evaluation Criteria in Solid Tumors; *ORR*, objective response rate; *DCR*, disease control rate; *CI*, confidence interval

^a^ORR was defined as the proportion of patients with a best overall tumor response, CR, or PR

^b^DCR refers to the proportion of patients with response and stable disease

**Fig. 4:**
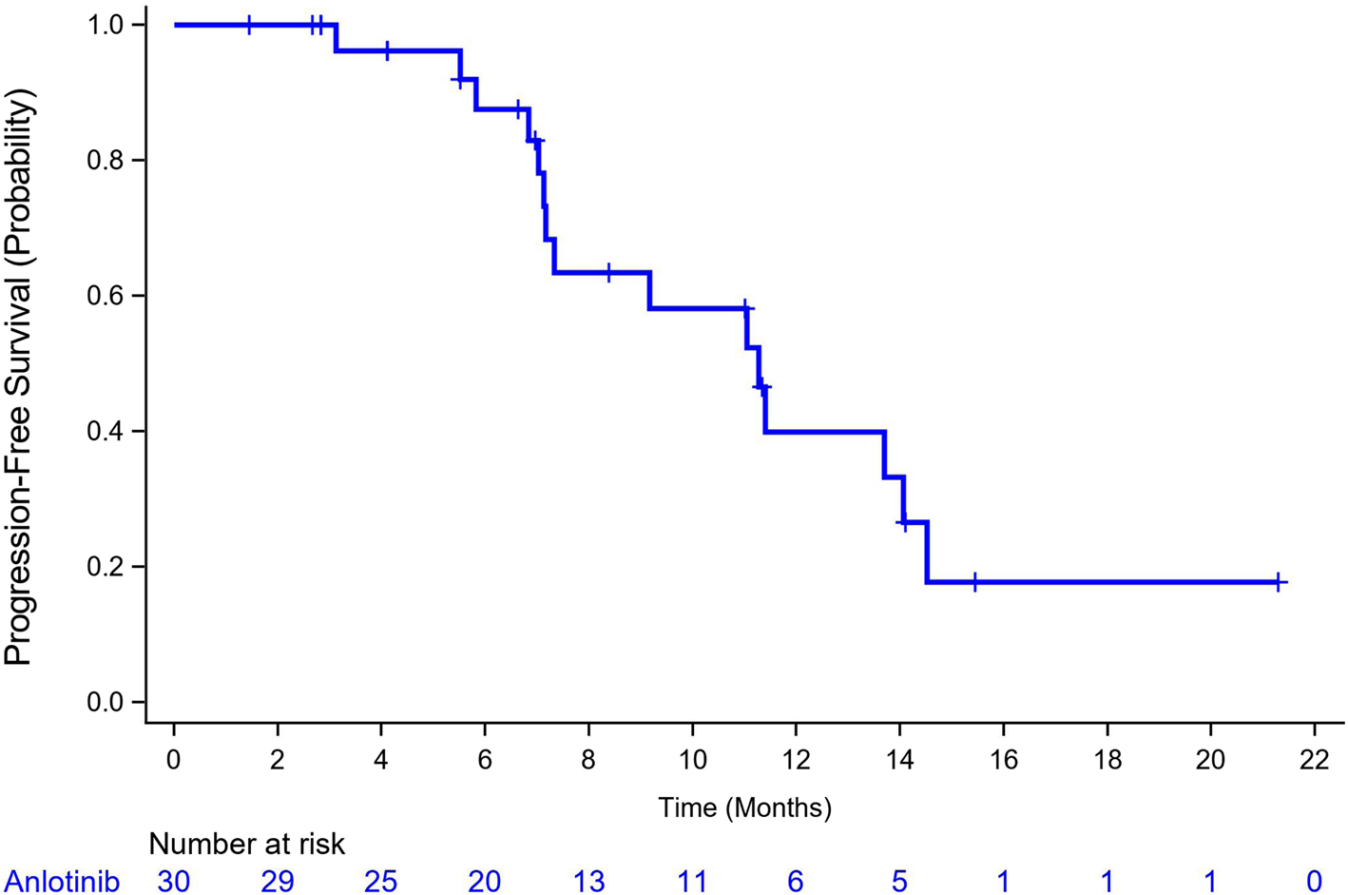
Estimates of the progression-free survival using Kaplan-Meier analysis (full analysis set, *N* = 30)

The median PFS according to Kaplan–Meier estimations was 11.3 months (95% CI, 7.1–14.1) (Fig. 4). The median PFS in responders (*n* = 23) was 13.7 months (95% CI, 7.2–14.5), while those with stable and progressive disease (*n* = 6) had a median PFS of 7.3 months (95% CI, 3.1–11.4). The PFS of maintenance treatment was also analyzed, which was named as PFS2 and defined as the time from maintenance treatment to disease progression. The median PFS2 was 7.1 months (95% CI, 4.5–9.8).

**Fig. 5:**
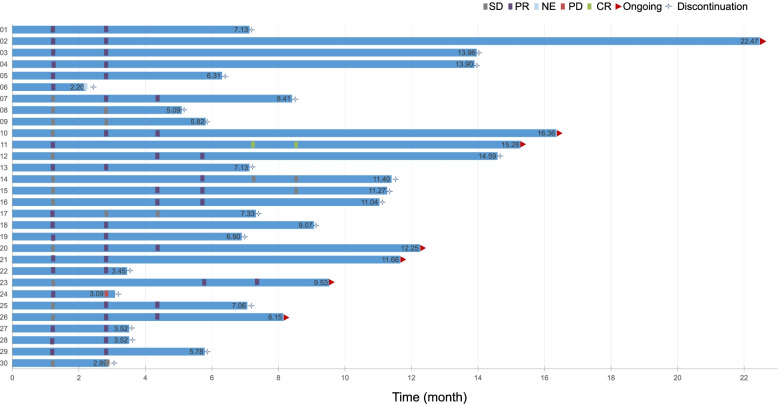
Swimmer plots of patients. Patients who received anlotinib plus oxaliplatin and capecitabine as first-line therapy in RAS/BRAF wild-type unresectable mCRC

The median DOR was 7.9 months (95% CI, 5.5–12.7). Despite the fact that it was not a pre-defined outcome, we assessed the investigator-reported DOT. The median DOT was 7.9 months (95% CI, 6.3–11.4) and 1 patient has received this regimen for at least 22 months (Fig. 5). The median duration of maintenance treatment was 6.9 months (95% CI, 3.0–9.8) (Table 3).

**Table 3:** Treatment duration and maintenance treatment duration

Treatment duration	*N* = 30
Months, median (95% CI)	7.9 (6.3–11.4)
Cycles, median (range)	11 (3–32)
Patients on treatment >6 months, *n* (%)	21 (70)
Patients on treatment >1 year, *n* (%)	7 (23.3)
**Maintenance treatment duration**	*N* = 24
Months, median (95% CI)	6.9 (3.0–9.8)
Patients on maintenance treatment >6 months, *n* (%)	11 (36.7)
Patients on maintenance treatment >1 year, *n* (%)	2 (6.7)

By post hoc analysis, factors including primary tumor location, primary tumor resected, metastatic sites, and serum carcinoembryonic antigen (CEA) level before chemotherapy were not associated with PFS.

### Safety

At least 1 treatment-emergent adverse event (TEAE) occurred in each of the 30 patients receiving the experimental treatment over the course of the trial. The most frequent TEAEs (≥10%) were presented in Table 4. A total of 25 (83.3%) patients experienced grade 3 or 4 TEAEs. Grade 5 TEAEs were not reported. The most frequent grade 3 or 4 TEAEs (≥10%) were hypertension (15/30; 50%), neutrophil count decreased (8/30; 26.7%), diarrhea (4/30; 13.3%), aspartate aminotransferase increased (3/30; 10.0%), platelet count decreased (3/30; 10%), hypertriglyceridemia (3/30,10%), and palmar-plantar erythrodysesthesia syndrome (3/30; 10%). The most common anlotinib-related TEAE of hypertension can be controlled by optimal management and does not lead to dose reduction. The peripheral neurotoxicity and hematotoxicity could be ascribed to oxaliplatin, while palmar-plantar erythrodysesthesia syndrome was the most frequent AE due to capecitabine. Only 1 case required anlotinib interruption due to the palmar-plantar erythrodysesthesia syndrome. Serious TEAEs (SAEs) including abnormal liver function, acute appendicitis, electrolyte disturbance, bone marrow suppression, intestinal obstruction, and colonic perforation occurred in 6 (20.0%) patients, while 4 patients (13.3%) were reported to have experienced drug-related SAEs. Four patients’ SAEs alleviated or recovered, while the others did not.

**Table 4:** Treatment-emergent adverse events occurring in ≥10% of patients (safety population, *N* = 30)

TEAE, *n* (%)	Grade 1	Grade 2	Grade 3	Grade 4	Any grade
Hypertension		11 (36.7%)	15 (50%)		26 (86.7%)
Palmar-plantar erthrodysesthesia syndrome	10 (33.3%)	9 (30%)	3 (10%)		22 (73.3%)
White blood cell decreased	12 (40%)	7 (23.3%)	2 (6.7%)		21 (70%)
Neutrophil count decreased	7 (23.3%)	5 (16.7%)	8 (26.7%)		20 (66.7%)
Vomiting	11 (36.7%)	2 (6.7%)	2 (6.7%)		15 (50%)
Decreased appetite	11 (36.7%)	4 (13.3%)			15 (50%)
Nausea	11 (36.7%)	2 (6.7%)			13 (43.3%)
Malaise	11 (36.7%)	1 (3.3%)			12 (40%)
Platelet count decreased	7 (23.3%)	2 (6.7%)	1 (3.3%)	2 (6.7%)	12 (40%)
Diarrhea	4 (13.3%)	2 (6.7%)	4 (13.3%)		10 (33.3%)
Peripheral neurotoxicity	8 (26.7%)	1 (3.3%)	1 (3.3%)		10 (33.3%)
Alanine aminotransferase increased	5 (16.7%)	3 (10%)	1 (3.3%)		9 (30.0%)
Aspartate aminotransferase increased	4 (13.3%)	2 (6.7%)	3 (10.0%)		9 (30.0%)
Constipation	4 (13.3%)	2 (6.7%)			6 (20%)
Toothache	5 (16.7%)	1 (3.3%)			6 (20%)
Fever	5 (16.7%)				5 (16.7%)
Abdominal pain	3 (10%)	1 (3.3%)	1 (3.3%)		5 (16.7%)
Thyroid-stimulating hormone increased	3 (10%)	1 (3.3%)			4 (13.3%)
Hypertriglyceridemia	1 (3.3%)		1 (3.3%)	2 (6.7%)	4 (13.3%)
Dizziness	4 (13.3%)				4 (13.3%)
Proteinuria	2 (6.7%)	1 (3.3%)			3 (10%)
Hypokalemia	2 (6.7%)			1 (3.3%)	3 (10%)
Hyponatremia	1 (3.3%)	1 (3.3%)	1 (3.3%)		3 (10%)
Cough	2 (6.7%)	1 (3.3%)			3 (10%)
Upper respiratory infection	3 (10%)				3 (10%)
Weight loss	1 (3.3%)	2 (6.7%)			3 (10%)
Headache	3 (10%)				3 (10%)

A total of 18 (60%) patients had TEAEs that resulted in dose reduction, interruptions, or delays. Five patients discontinued therapy at the induction stage due to TEAEs. Reasons for discontinuations were abnormal liver function (grade 4), bone marrow suppression (grade 3), palmar-plantar erythrodysesthesia syndrome (grade 2), and colonic perforation (grade 4).

During the study, 4 patients underwent surgery after anlotinib administration, including 2 patients who underwent surgery for intestinal obstruction after anlotinib discontinuation for 7 or 10 days, and the other 2 received radical surgery considering the benefits of patients after anlotinib discontinuation for 8 or 10 days. No bleeding and anastomotic fistula were observed during the perioperative period.

## Discussion

The ALTER-C-002 study reached its primary endpoint, with patients treated with anlotinib, oxaliplatin, and capecitabine showing an improved ORR compared with those receiving conventional treatment. It had been seen that the ORR of chemotherapy alone was 35%–55% while the ORR in this trial was 76.7% [26, 27]. The addition of anlotinib to oxaliplatin and capecitabine resulted in tumor shrinkage in all patients. Although it was a non-head-to-head study, anlotinib combined with oxaliplatin and capecitabine therapy correlated with a longer median PFS (11.3 months) when compared with CAPEOX alone (8.0 months) [26, 27], suggesting synergistic action of anlotinib, oxaliplatin, and capecitabine.

Moreover, the ORR and median PFS in our study were comparable to historical data from patients with RAS/BRAF wild-type disease who received chemotherapy in combination with bevacizumab (ORR 55.2%; median PFS 10.6 months) or cetuximab (ORR 59.6%; median PFS 10.5 months) [28].

The ALTER-C-002 study was designed in such a way that any component of the therapy might be modified in order to manage AEs. Under the guidance of this customized strategy, we discovered that this combined regimen seemed to have a manageable tolerability profile when used as first-line therapy for patients with RAS/BRAF wild-type mCRC. Overall, the AE profile of the combined anlotinib, oxaliplatin, and capecitabine therapy seemed to be perfectly compatible, without unexpected AEs. Moreover, the incidence of palmar-plantar erythrodysesthesia syndrome did not increase when anlotinib was added. Besides chemotherapy-related TEAEs, some anlotinib-related TEAEs, including hypertension and proteinuria, were identified. In our study, we reduced the dosage of capecitabine to 850 mg/m^2^ to minimize palmar-plantar erythrodysesthesia syndrome. Ten patients required dose reduction of capecitabine for TEAEs like palmar-plantar erythrodysesthesia syndrome, platelet count decreased, abnormal liver function, and weight loss. Besides, 10 patients took oxaliplatin dose reduction due to peripheral neurotoxicity, hematotoxicity, vomiting, weight loss, and abnormal liver function. Four patients required dose reduction of anlotinib due to palmar-plantar erythrodysesthesia syndrome, platelet count decreased, gum bleeding, and proteinuria.

Interestingly, although the treatment duration was not a pre-specified endpoint, 7 patients received the study medication for longer than 1 year, with the median DOT of 7.9 months (95% CI, 6.3–11.4). Moreover, 1 patient has received this regimen for at least 22 months. The extended DOT reported, even though in a phase II research, differed from findings reported in phase III trials of other first-line treatments for mCRC, where the DOT was seldom longer than 6 months [26, 27].

Maintenance treatment is very important after the initiation of induction therapy, and a variety of drugs have been evaluated for maintenance therapy. Although progress has been made in recent years, the best maintenance regimen that balances effectiveness with safety and costs has not been developed. Following completion of a phase III study, it was discovered that maintenance treatment with a single agent of capecitabine might be an effective treatment alternative since the PFS in the capecitabine maintenance group was statistically significantly prolonged as opposed to that in the observation group (6.43 months versus 3.43 months; HR = 0.54, *P* < 0.001) [29]. Comparing the current trial to earlier research, the median PFS2 was 7.1 months, which is comparable to fluoropyrimidine with bevacizumab (6.3 months) or capecitabine with bevacizumab (8.5 months) [30, 31]. However, the difference is that anlotinib is an oral, small molecule, multi-target TKI. The participants could avoid central venous catheterization for the treatment, which not only reduced a series of catheter-related complications [32, 33], such as venous thrombosis and infection, but also reduced the length of stay in treatment facility. Moreover, during maintenance treatment, patients do not need any intravenous infusion, which heavily improved patients’ medical compliance and quality of life, especially in the Covid-19 era. In the study, 13 patients received FOLFIRI plus cetuximab therapy after PD. Among these patients, all received more than one tumor evaluation, with 8 PR, 3 SD, and 2 PD cases. This indicated that anlotinib usage does not influence the efficacy of cetuximab plus chemotherapy, which may benefit the OS.

The ALTER-C-002 trial has several limitations, including a single-arm design, a limited sample size, and the absence of an OS analysis. In addition, this non-global trial was only carried out in Zhejiang province in China. In spite of the limitations, the ORR and PFS in this research were substantially better than those obtained in regimens incorporating other TKIs [34–37]. It may be possible to determine whether this theorized effect translates into prolonged OS once data are available from longer-term follow-up of patients in ALTER-C-002.

## Conclusions

In conclusion, anlotinib combined with oxaliplatin and capecitabine achieved considerable ORR, DCR, and PFS and showed potential efficacy as first-line therapy for mCRC with manageable toxicity profiles. No new safety signals were identified with anlotinib when combined with capecitabine and oxaliplatin. Our findings could also provide a framework for additional insight into the application of anlotinib with capecitabine as a maintenance medication in patients with RAS/BRAF wild-type mCRC who have obtained clinical benefits from induction therapy. The efficacy of this treatment is comparable to standard treatment in mCRC. We are launching a randomized, multicenter, phase 3 trial (NCT04854668) to further assess the efficacy of this regimen.

## Data Availability

The datasets generate and analyzed during the current study are available from the corresponding author on reasonable request.

## Abbreviations

CEA: Carcinoembryonic antigen
CI: Confidence interval
CRC: Colorectal cancer
CR: Complete response
DCR: Disease control rate
DOR: Duration of response
DOT: Duration of treatment
ECOG PS: Eastern Cooperative Oncology Group performance status
FAS: Full analysis set
FGFR: Fibroblast growth factor receptor
KM: Kaplan-Meier
mCRC: Metastatic CRC
NE: Inevaluable
NCI CTC AE: National Cancer Institute Common Terminology Criteria for Adverse Events
OS: Overall survival
ORR: Objective response rate
PD: Progressive disease
PDGFR: Platelet-derived growth factor receptor
PFS: Progression-free survival
PR: Partial response
RECIST: Response Evaluation Criteria in Solid Tumors
SAEs: Serious TEAEs
SD: Stable disease
SAS: Safety analysis set
TKIs: Tyrosine kinase inhibitors
TEAE: Treatment-emergent adverse event
VEGFR: Vascular endothelial growth factor receptor
